# A novel surgical management for pediatric patients with irreducible atlantoaxial dislocation: Transoral intraarticular cage distraction and fusion with C-JAWS staple fixation

**DOI:** 10.3389/fsurg.2022.1054695

**Published:** 2023-01-06

**Authors:** Xiaobao Zou, Haozhi Yang, Suochao Fu, Chenfu Deng, Junlin Chen, Rencai Ma, Xiangyang Ma, Hong Xia

**Affiliations:** Department of Orthopedics, General Hospital of Southern Theatre Command of PLA, Guangzhou, China

**Keywords:** irreducible atlantoaxial dislocation, transoral approach, reduction, internal fixation, spinal fusion

## Abstract

**Background:**

Currently, irreducible atlantoaxial dislocation (IAAD) can be treated by a single transoral approach in one stage to reduce surgical injuries to patients. However, the widely used fixation devices are not suitable for pediatric patients because of larger profile of devices.

**Objective:**

The purpose of this study is to report the preliminary clinical outcomes of a novel surgical technique by transoral intraarticular cage distraction and fusion with C-JAWS staple fixation for pediatric patients with IAAD.

**Methods:**

From June 2011 to June 2014, eight pediatric patients with IAAD were enrolled and treated by this technique in our department. Patients' clinical data were retrospectively analyzed, including neurological status, clinical symptoms, reduction, bone fusion, and complications.

**Results:**

The surgeries were successfully performed in all patients without injuries to spinal cord, nerve and blood vessel. Clinical symptomatic relief was presented on all 8 patients (100%). Satisfactory reduction was indicated by significant decrease of atlanto-dental interval postoperatively (*P* < 0.05). The remarkable improvement of postoperative neurological function has been proved by significant increase of Japanese Orthopaedic Association score (*P* < 0.05). The average follow-up duration was 19.4 ± 5.8 months (range 12–30 months). Bone fusion was achieved in all 8 cases. No complications were documented after operation and during follow-up.

**Conclusions:**

Transoral intraarticular cage distraction and fusion with C-JAWS staple fixation is an effective treatment for pediatric patients with IAAD, which can achieve satisfactory reduction, fixation and bone fusion.

## Introduction

Atlantoaxial dislocation is a common disease in the craniocervical junction, which can be caused by inflammation, tumor, trauma, congenital malformation, degeneration and other factors. This disease can cause neck pain, numbness and weakness in the limbs, hemiplegia and other symptoms, and can be life-threatening in severe cases (1). According to Yin et al.'s classification system, the atlantoaxial dislocation is divided into reducible dislocation, irreducible dislocation and fixed dislocation based on the degree of difficulty in reducing the dislocation, which determines the surgical choices (2).

Surgical treatment of irreducible atlantoaxial dislocation (IAAD) commonly requires transoral release plus posterior reduction and fixation (3). For pediatric patients with IAAD, more injuries may be caused by anteroposterior surgery. After performing transoral anterior release, the position change from supine to prone would increase the risk of spinal cord injury due to extreme atlantoaxial instability (4). Anteroposterior surgery can result in more soft tissue damage and bleeding. Currently, the single transoral approach can achieve release, decompression, reduction, fixation and fusion for IAAD in one stage (5). However, the present fixation devices, like the well-known transoral atlantoaxial reduction plate (TARP) (6), have a larger shape, which is not suitable for pediatric patients in most situations (4). The C-JAWS, a cervical compressive staple, with a smaller shape, has been used in anterior cervical discectomy and fusion (ACDF) but not in the atlantoaxial joint. In this study, a novel surgical technique by transoral intraarticular cage distraction and fusion with C-JAWS staple fixation was performed in 8 pediatric patients with IAAD, and the clinical data were retrospectively analyzed to evaluate the clinical effects of this technique.

## Materials and methods

### Patients

From June 2011 to June 2014, a total of 8 pediatric patients (3 boys and 5 girls, 10.0 years old on average, range 8–12) with IAAD underwent transoral surgeries using an intraarticular cage and C-JAWS staple (Table 1). The average disease duration was 10.5 ± 6.6 months (range 3–24 months). The clinical symptoms were as follows: progressive extremity numbness (8/8, 100%), extremity weakness (6/8, 75.0%), occipitocervical pain (5/8, 62.5%), unsteady gait (2/8, 25.0%), and hemiparesis (1/8, 12.5%) (Table 2).

**Table 1:** Pre- and postoperative data of the 8 patients.

Case	Age (year)/Sex	Duration of symptom (month)	ADI (preop)	ADI (postop)	JOA (preop)	JOA (postop)	Bone fusion confirmed (month)	Follow-up (month)	Complication
1	12/M	24	8.5	2.1	11	15	6	15	No
2	10/F	15	7.3	1.5	10	14	6	18	No
3	8/M	8	9.8	2.5	8	12	3	24	No
4	9/F	6	6.4	0.5	8	14	6	30	No
5	12/F	3	7.1	1.0	10	15	3	20	No
6	11/M	12	8.2	1.5	9	12	6	12	No
7	10/F	6	5.2	0	11	15	3	15	No
8	8/F	10	6.6	0.8	13	16	6	21	No
M ± SD		10.5 ± 6.6	7.4 ± 1.4	1.2 ± 0.8	10.0 ± 1.7	14.1 ± 1.5		19.4 ± 5.8	
T			27.025		−11.773				
*P*			0.000^a^		0.000^a^				

F, female; M, male; ADI, atlanto-dental interval; JOA, Japanese Orthopedic Association score; M ± SD, mean ± standard deviation.

^a^ Paired-sample *t*-test.

**Table 2:** Clinical symptoms.

Symptoms	Preoperative no. (%)	Postoperative improvement no. (%)
Occipitocervical pain	5 (62.5%)	5 (100%)
Extremity numbness	8 (100%)	7 (87.5%)
Extremity weakness	6 (75.0%)	5 (83.3%)
Unsteady gait	2 (25.0%)	2 (100%)
Hemiparesis	1 (12.5%)	1 (100%)

Each patient may have one or more symptoms.

### Preoperative examinations

Before surgery, plain cervical radiographs, computed tomography (CT) scans and magnetic resonance imaging (MRI) were performed for all patients. Two cases were complicated with old odontoid fracture, 4 cases with basilar invagination, and 2 cases had undergone posterior surgery. All patients have undergone skull traction in a hyperextended position for one week. The weight of skull traction is 1/12 to 1/10 of body weight. All 8 cases were diagnosed as IAAD, for which AAD could not be restored by traction. MRI showed obvious compression of the cervical cord in all of the cases. The average Japanese Orthopaedic Association (JOA) score (17-point system) was 10.0 ± 1.7, and atlanto-dental interval (ADI) was 7.4 ± 1.4 mm.

### Surgical procedure

Preoperative preparations: An oral examination and dental cleaning were performed before surgery. Oral cleaning with 0.02% vinegar chlorhexidine was performed 3–6 times per day for 3 days before surgery. Broad-spectrum antibiotics were administered intravenously 30 min before surgery.

**Figure 1:**
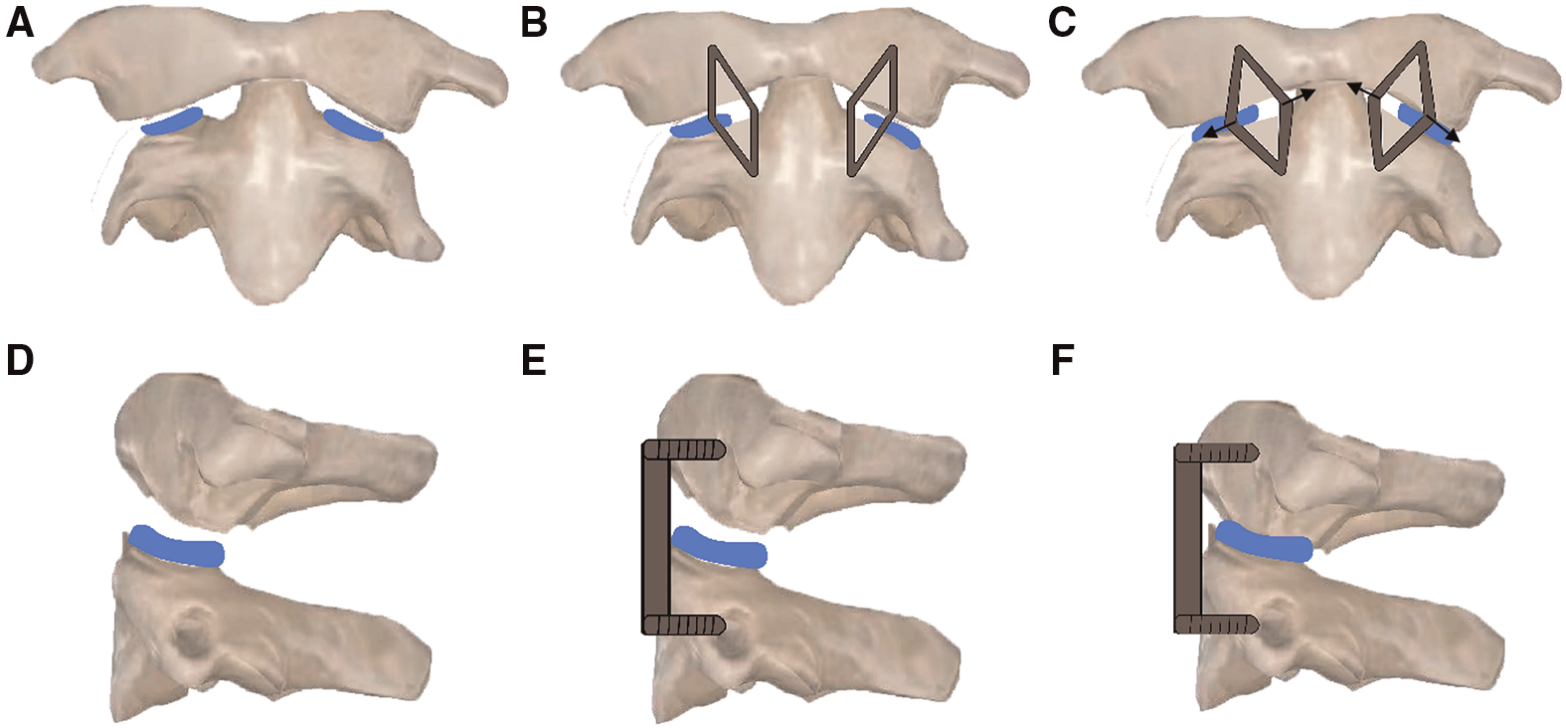
Schematic diagram of operation. (**A,D**) Two intraarticular cages were inserted into the bilateral lateral mass joints. (**B,E**) Two C-JAWS staples were fixed to the atlantoaxial joint, with the cephalad end fixed into the C1 lateral mass and the caudal end fixed into the C2 vertebral body. (**C,F**) Two C-JAWS staples were spreaded to both sides to produce compression on joints.

**Figure 2:**
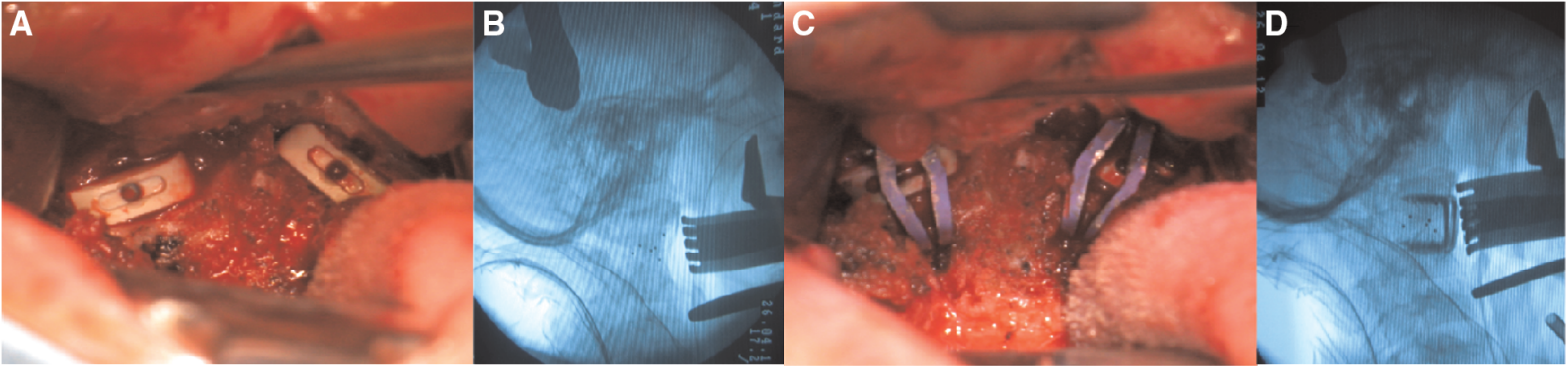
Intraoperative procedures. (**A,B**) The bilateral intraarticular cages were implanted and intraoperative fluoroscopy showed satisfactory atlantoaxial reduction. (**C,D**) Two C-JAWS staples were fixed and intraoperative fluoroscopy showed satisfactory location of holders.

Surgical techniques: Under general anaesthesia with nasotracheal intubation, the patient was placed in supine position with skull traction of 4–6 kg. After disinfection of the oral cavity, a middle longitudinal incision was made in the posterior pharyngeal wall. The mucosa and muscle were then separated to expose the C1 anterior arch, C2 vertebral body and bilateral lateral mass joints. Then, the anterior scar tissue and hyperplastic callus between the odontoid and anterior arch were resected. After incision of the capsules of bilateral lateral mass joints, the intraarticular adherent tissues and articular cartilage were removed with a curette and grinding drill to completely loosen the atlantoaxial joint. Two intraarticular cages (Wego, Shandong, China) filled with autologous iliac bone were then inserted into the bilateral lateral mass joints for distraction and bone fusion (Figures 1A,D, 2A). Afterwards, reduction of the atlantoaxial joint and well-placement of cages were identified by intraoperative x-ray (Figure 2B), two appropriate C-JAWS staples (Medicrea, Lyon, France) were used to fix the atlantoaxial joint, with the cephalad end fixed into the C1 lateral mass and the caudal end fixed into the C2 vertebral body (Figures 1B,E), and a slight compression was applied at the holders by spreading it to both sides (Figures 1C,F, 2C). The length of the nail portion of the C-JAWS staple is 12–18 mm. Afterwards, reduction of the atlantoaxial joint and the desired position of implantation were further confirmed by intraoperative x-ray (Figure 2D). Eventually, the wound was closed in layers.

### Postoperative management and follow-up

The nasal trachea cannula was removed in 24–48 h postoperatively, and the nasogastric tube was removed on day 7 postoperatively. Ultrasonic nebulisation and 0.02% chlorhexidine acetate gargling were performed 3–6 times per day for 7 days. Broad-spectrum antibiotics were administered introvenously for 3 days. The cervical x-ray, CT scan and MRI were performed postoperatively. The ADI was measured to evaluate the reduction of C1–C2. The patients' neurological status was assessed using the JOA score. Bone fusion was confirmed by CT scan. All patients were asked to wear a rigid Philadelphia cervical collar for 3 months and were followed up at 3, 6, 9 and 12 months and then once per year. If bone fusion was not achieved, patients needed to keep wearing the cervical collar until confirmation of bone fusion.

### Statistical analysis

SPSS 21.0 software (IBM, Armonk, NY, United States) was used for the statistical analysis. The K-S test was used to verify the normal distribution of data. All data were expressed as mean and standard deviation. ADI and JOA scores before and after surgery were compared using paired-sample *t*-test, and *P*-value <0.05 was considered statistically significant.

## Results

**Figure 3:**
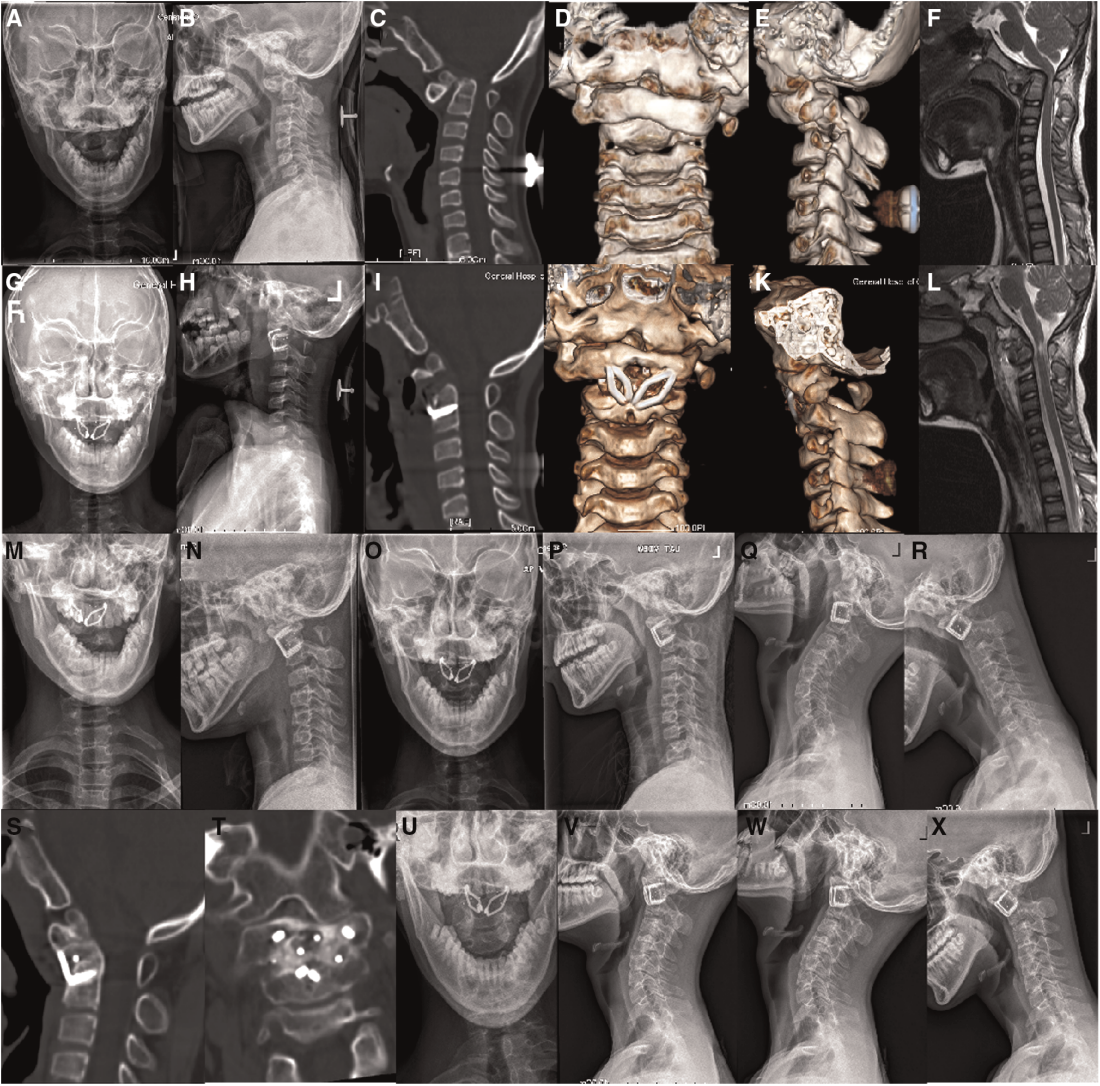
A 12-year-old boy, who was diagnosed IAAD with old odontoid fracture, underwent transoral intraarticular cage distraction and fusion with C-JAWS staple fixation. (**A–E**) Preoperative cervical x-rays and CT scans with three-dimensional reconstruction showed evidence of IAAD with old odontoid fracture. (**F**) Preoperative Sagittal MRI revealed compression of the spinal cord. (**G–K**) Cervical x-rays and CT scans with three-dimensional reconstruction performed at 1 week after revision surgery showed satisfactory reduction and good placement of fixation and cages. (**L**) Postoperative sagittal MRI showed a desirable decompression of the spinal cord. (**M,N**) Cervical x-rays at 3-month follow-up showed stable fixation. (**O–R**) Cervical x-rays at 6-month follow-up showed stable fixation without loss of reduction. (**S,T**) CT scans at 6-month follow-up revealed a solid bone fusion. (**U–X**) Cervical x-rays at last follow-up showed good C1–C2 sequence.

Surgeries on 8 cases were performed successfully. The average operative time was 152.5 ± 32.0 min (range 110–200 min), with average intraoperative blood loss of 77.5 ± 22.5 ml (range 50–110 ml). No spinal cord, vascular or duramater injuries occurred during the operation. Clinical symptoms were relieved in all patients (Table 2). The average follow-up time was 19.4 ± 5.8 months (range 12–30 months). Satisfactory reduction of C1–C2 were achieved in all cases shown on postoperative radiographs and CT scans, with a marked reduction of postoperative ADI (1.2 ± 0.8 mm) compared to preoperative ADI (7.4 ± 1.4 mm, *P* < 0.05). Decompression of spinal cord were found on postoperative MRI (Figure 3). Postoperative neurological function was significantly improved, with significant improvement of JOA score from preoperative 10.0 ± 1.7 to postoperative 14.1 ± 1.5 (*P* < 0.05). All of the cases obtained bone fusion in 3–6 months after operation. No complications of re-dislocation or neurological deterioration were documented after operation and during the follow-up.

## Discussion

IAAD usually results in spinal cord compression and profound neurologic deficits. Therefore, a surgical therapy is imperative to obtain symptoms alleviation and spinal cord decompression (1). It is a common view that transoral release plus posterior reduction, fixation and fusion is necessary for surgical treatment of IAAD (3, 7, 8). But an anteroposterior approach can cause more surgical trauma, especially performed on pediatric patients. Moreover, after performing transoral release, the risk of spinal cord injury would be increased when changing patient's posture because of the extremely unstable atlantoaxial joint (9).

Currently, a single transoral approach can achieve release, decompression, reduction, fixation and fusion for IAAD in one stage to reduce surgical trauma to patients (5). The transoral atlantoaxial reduction plate (TARP), a well-known transoral technique, designed by our institution in 2004, can achieve release, reduction, decompression, fixation and fusion in one stage through a single transoral approach, that provides an effective surgical approach for the treatment of IAAD accompanied by spinal cord compression (5, 6, 10, 11). But, according to our clinical experience, the thicker thickness and large shape of the TARP make it difficult to conveniently accomplish the surgical procedures in most pediatric patients with limited oral space and smaller anatomical structure (4). Additionally, the insufficient soft tissues of the pharyngeal wall to cover the plate potentially leads to the occurrence of postoperative dysphagia and disruption of wound (5, 6).

The C-JAWS, a cervical compressive staple, has a thinner thickness of 1.5 mm and smaller shape than common anterior cervical plate, and has been commonly used for intervertebral compression fixation after implantation of interbody cage in ACDF. Fiere et al. (12) reported a dependable biomechanical stability of C-JAWS staple in a vitro testing and the early clinical results of 23 cases who underwent ACDF using an interbody cage and C-JAWS staple showed various advantages including short incision, short operative time and lower rate of dysphagia incidences as compared to most of the anterior cervical plate. Xia et al. (13) presented the similar result of 9 cases who underwent ACDF with an interbody cage and C-JAWS staple. The authors believed that the usage of the C-JAWS staple when performing transoral fixation after implantation of intraarticular cage to atlantoaxial joint would be simplify the surgical procedure, reduce the wound tension and operative trauma, which benefited from its thinner and smaller shape.

In this study, we reported a novel surgical technique by transoral intraarticular cage distraction and fusion with C-JAWS staple fixation in 8 pediatric patients with IAAD. The C-JAWS staple was used to stabilize bilateral lateral mass joints of C1–C2 after placement of intraarticular cage and evaluated the clinical effects. All patients achieved satisfactory reduction, reliable fixation, improvement of neurological function and bone fusion without complications during the operation and the follow-up.

### Limitations

Several limitations in the current study should be noted. Firstly, the sample size is ratter small. With larger cases performed on this technique, the clinical efficacy may be more thoroughly evaluated. Secondly, this is a retrospective study. Further prospective studies need to better control the follow-up intervals and require more standardized measurements.

## Conclusion

Transoral intraarticular cage distraction and fusion with C-JAWS staple fixation is an effective and safe surgical option to treat IAAD in pediatric patients. The use of intraarticular cage can distract the atlantoaxial joint to obtain satisfactory reduction and facilitate bone fusion, and the C-JAWS staple can provide reliable fixation, that offers a new method for anterior atlantoaxial fixation through a transoral approach in pediatric patients.

## Data Availability

The raw data supporting the conclusions of this article will be made available by the authors, without undue reservation.
